# Toward the Enhancement of Microalgal Metabolite Production through Microalgae–Bacteria Consortia †

**DOI:** 10.3390/biology10040282

**Published:** 2021-04-01

**Authors:** Lina Maria González-González, Luz E. de-Bashan

**Affiliations:** 1 The Bashan Institute of Science, 1730 Post Oak Ct, Auburn, AL 36830, USA; lina@bashanis.org; 2 Environmental Microbiology Group, Northwestern Center for Biological Research (CIBNOR), Avenida IPN 195, La Paz, Baja California Sur 23096, Mexico; 3 Department of Entomology and Plant Pathology, Auburn University, 209 Life Sciences Building, Auburn, AL 36849, USA

**Keywords:** mutualism, microalgae, growth-promoting bacteria, metabolites, biorefinery

## Abstract

**Simple Summary:**

Microalgae are photosynthetic microorganisms with high biotechnological potential. However, the sustainable production of high-value products such as lipids, proteins, carbohydrates, and pigments undergoes important economic challenges. In this review, we describe the mutualistic association between microalgae and bacteria and the positive effects of artificial consortia on microalgal metabolites’ production. We highlighted the potential role of growth-promoting bacteria in optimizing microalgal biorefineries for the integrated production of these valuable products. Besides making a significant enhancement to microalgal metabolite production, the bacterium partner might assist in the biorefinery process’s key stages, such as biomass harvesting and CO_2_ fixation.

**Abstract:**

Engineered mutualistic consortia of microalgae and bacteria may be a means of assembling a novel combination of metabolic capabilities with potential biotechnological advantages. Microalgae are promising organisms for the sustainable production of metabolites of commercial interest, such as lipids, carbohydrates, pigments, and proteins. Several studies reveal that microalgae growth and cellular storage of these metabolites can be enhanced significantly by co-cultivation with growth-promoting bacteria. This review summarizes the state of the art of microalgae–bacteria consortia for the production of microalgal metabolites. We discuss the current knowledge on microalgae–bacteria mutualism and the mechanisms of bacteria to enhance microalgae metabolism. Furthermore, the potential routes for a microalgae–bacteria biorefinery are outlined in an attempt to overcome the economic failures and negative energy balances of the existing production processes.

## 1. Introduction

Microalgae are photosynthetic microorganisms with a high potential to produce a wide variety of industrial-interest metabolites such as proteins, lipids, carbohydrates, and pigments. Their rapid growth rates and the possibility to be cultivated on nonarable land constitute an advantage against plant-based sources [1].

Overproduction of microalgal metabolites has been optimized by several mechanisms, from the alteration of culture conditions (nutrient concentrations, light intensity, carbon source, salinity, and temperature) to metabolic engineering [2,3,4,5]. Another way to stimulate metabolite production is through the application of chemical triggers, such as phytohormones and analogs regulating microalgal metabolism, or of chemicals that can regulate biosynthetic pathways, induce oxidative stress responses, or act directly as metabolic precursors [6,7,8]. Since these growth-promoting factors are produced by bacteria, microalgae–bacteria consortia have been explored as an alternative route to enhancing microalgae growth and metabolite production [9,10]. 

Microalgae and bacteria have coexisted since the early stages of evolution. From a biotechnological aspect, the symbiotic interactions cover a wide range, mostly mutualistic, commensalistic, or parasitic [11]. Mutualisms are positive interactions among different species that improve the fitness of the involved partners and are based on the exchange of resources and services [12,13]. A mutualistic microalga–bacteria consortium is based on the exchange of metabolites, mostly the bacterial uptake of extracellular organic carbon released from the algal photosynthesis; in return, the bacterial growth can be stimulated by (1) the removal of oxygen and the generation of carbon dioxide, (2) the supply of nutrients, vitamins, and trace elements for microalgal growth, and (3) the production of growth-promoting factors as well as chelators and phytohormones [14]. 

In this review, we initially discuss how a mutualistic microalga–bacteria consortia is established; then, we summarize the current studies that address the positive effects of the consortia on microalgal metabolites’ production of commercial interest such as proteins, lipids, carbohydrates, and pigments. Furthermore, we discuss the potential routes that can be adopted for a sustainable and cost-efficient valorization of the biomass through the integrated production of microalgal metabolites in microalgae–bacteria biorefineries, highlighting the main stages that can be optimized.

## 2. Mutualistic Interaction in Microalgae–Bacteria Consortia

### 2.1. The Concept of Phycosphere 

Algae are responsible for nearly 50% of the world’s atmospheric carbon fixation, and more than half of the algae are dependent on interaction with bacteria for supplying essential micronutrients [15]. An important concept in the interaction between microalgae and bacteria is the “phycosphere,” a sheath surrounding the microalgal cells, composed mainly of carbohydrates and proteins, where each group influences the other in a stimulatory and inhibitory fashion [16,17,18]. The physical dimensions of the phycosphere are probably transitory and depend upon the concentration and rate of release of materials as well as on the degree of micro and macro turbulence in the environment. Within the phycosphere, the mechanisms of interactions between bacteria and microalgae are diverse and involve specific cellular processes and communication such as attachment and quorum sensing [19].

According to Kouzuma and Watanabe [20], the mutualistic interactions between microalgae and bacteria are of three types: nutrient exchange, signal transduction, and gene transfer. In general, nutrient exchange has been considered the most common and important. Microalgae exude a part of the photosynthesized organics as dissolved organic carbon (DOC); these are assimilated by heterotrophic bacteria, and in return, bacteria contribute to algal growth through the generation of CO_2_, micronutrients (such as Fe), vitamins, macronutrients, specifically fixed N and phytohormones [15,17]. The affinity of bacteria to DOC and their growth kinetics are key parameters in the interaction with the microalgae [20]. 

In a study that analyzed the phycosphere of microalgae, including *Guinardia delicatula*, *Pseudo-nitzschia pungens*, *Thalassiosira rotula*, *Skeletonema costatum*, *Ceratium horridum*, and *Akashiwo sanguinea*, the associated bacterial communities were characterized by ribosomal intergenic spacer analysis and denaturing gradient gel electrophoresis coupled with the assessment of biotic and abiotic parameters [21]. The authors found that the composition of the bacterial communities was not strictly species-specific for the microalgae and could not be correlated with the growth of the microalgae or the depletion of inorganic nutrients from the growth medium. Thus, parameters such as quality and quantity of exudates, i.e., the composition of the DOC, must be considered. Notwithstanding this, using 454 pyrosequencing, Ramanan et al. [22] found that the major phylotypes of bacteria from green algae isolated from different habitats are similar, showing the limitation of fingerprinting methods to assess the composition of the community. However, the mechanisms involved in the interaction do seem to be species-specific, depending on the microenvironment and conditions of each microalga [11]. Clearly, the omics approaches and next-generation sequences allow an in-depth understanding of the microalgae–bacteria association [11]. Cooper and Smith [23] suggest the combined use of traditional methods, such as microbial and biochemical analysis, with the omics approaches. By a metabolomic analysis, it would be possible to detect the range of compounds that are exchanged between the partners, while the metagenomic and transcriptomic approaches would allow the ascertaining of the interactions, genomic machinery, and regulatory patterns.

Some microalgae show higher interaction with bacteria, probably due to an evolutionary predisposition. For example, by stable isotope tracing and nanoSIMS visualization, Samo et al. [17] quantified the cell–cell exchange of elements between the microalgae *Phaeodactylum tricornutum* and *Nannochloropsis salina,* with their associated bacteria. The authors reported that attachment defines a differential response, while 92–98% of cells of *P. tricornutum* had attached bacteria and fixed 64% more carbon than axenic cells. In contrast, only 42–63% of *N. salina* cells had associations with bacteria and fixed just 10% more carbon compared to axenic cells. In a mutualistic interaction, an uncultured bacterium related to *Haliscomenobacter* sp. increased carbon fixation in *P. tricornutum* and incorporated 71% more fixed C than other bacteria [17].

### 2.2. Mechanisms Acting in Mutualistic Co-Cultures

Most mutualistic interactions are inherently complicated by multiple variables, and therefore, it can be difficult to isolate their components for specific studies and determine if they are truly mutualistic [24]. However, through the use of two species or strains that do not interact in nature, it is possible to construct a synthetic mutualism [25]. The successful creation of mutualistic co-cultures is of great importance in synthetic biology, as multispecies consortia have a proven potential for biotechnological applications [26,27]. Nonetheless, the cultivation of several microorganisms is a complex procedure that can affect the outcome of the interaction; co-cultures can differ in parameters, such as the number of individual populations, the degree of separation between populations, the volume of cultures, and the timescale of the co-cultures. For example, cell–cell interactions are strongly influenced by the external environment, which is determined by the experimental setup. As a result, major considerations must be made with respect to foundational and mechanistic understanding when artificially constructing a mutualistic relationship system [26,27]. The inoculation ratios of the microorganisms play an important role in the performance of the synthetic consortium [28,29].

The experimental setup of the consortium can be of different types. The most common co-cultivation system is with both species on suspension [30,31,32]. Other ways of co-culture are by the immobilization of one of the microorganisms in biofilms while the other remains in suspension [33,34], with biofilms or different carriers embedding both microorganisms [35,36,37,38], or by the separation of the two species with a membrane [38,39]. Additionally, the growth-promoting effects of bacteria can also be evaluated on axenic microalgal cultures by cultivation with bacterial extracts [40,41] or through the addition of bacterial volatiles and gas exchange [42].

The mechanisms involved in a synthetic mutualistic interaction between microalgae and bacteria are similar to those found in natural associations. However, there is a bigger body of information regarding synthetic mutualisms, since cultivation under controlled laboratory or microcosm conditions involves fewer variables than experiments conducted in natural settings, and thus, they can be more straightforward to interpret [27]. For the purpose of this review, we mostly highlight synthetic mutualistic reports.

In co-cultures of diatoms and bacteria isolated from Lake Constance (Germany), it was found that growth and extracellular polymeric substances (EPS) secreted by the microalgae were regulated by the presence of the bacteria or their exudates [43]. For example, *Achnanthes minutissima*, *Pseudostaurosira* spp, and *P. tricornutum* experienced an increase of nearly 200% in chlorophyll-specific EPS concentration when the bacterial exudate was added to the culture. Searching for putative bacterial signal substances, the authors found that concentrations of various dissolved free amino acids within the diatom cultures changed drastically because of the interaction with the bacteria. In co-cultures of *P. tricornutum* and *Escherichia coli,* mass spectrometric peptide mapping allowed the identification of proteins possibly involved in signaling, extracellular carbohydrate modification and uptake, protein and amino acid modification, and cell–cell aggregation of diatoms and bacteria strains.

One of the main tradeoffs in the mutualistic interaction between microalgae and bacteria is the exchange of fixed carbon by microalgae and vitamins produced by the bacteria, which act as cofactors for enzymes in key metabolic pathways in the microalga [23]. Through a metagenomic analysis of algae-associated biofilms, Krohn-Molt et al. [44] found that genes involved in the biosynthesis of B vitamins are abundant and functional, suggesting their key roles in algae–bacteria association. Cooper and Smith [23] highlighted the fact that this trait is widespread in all algal lineages, indicating a possible environmental factor that drives the co-evolution between microalgae and bacteria. Nearly 50% of microalgae are auxotrophs for vitamin B12 (cobalamin) and 22% for vitamin B1 (thiamine). Thus, the most studied interaction is the one between B12 producer bacteria and cobalamin auxotrophs microalgae. As vitamin B12 is produced only by prokaryotes, it is the source of the vitamin for the microalga in exchange for photosynthates [23].

Croft et al. [15] found that the vitamin B12 producer *Halomonas* sp., isolated from non-axenic cultures of *Amphidinium operculatum,* can support the growth of the cobalamin-auxotroph *Porphyridium purpureum,* similarly to exogenous vitamin B12. The authors demonstrated that the role of the cobalamin in the algal metabolisms is a cofactor for cobalamin-dependent methionine synthase. Because the medium where these two microorganisms were growing did not contain any organic carbon source, it is logical to assume that the bacteria grow at the expense of the photosynthates produced by the microalga.

A model interaction for the exchange of vitamin B12 is the one created between the soil rhizobiaceae *Mesorhizobium loti* and the freshwater green microalga *Lobomonas rostrata,* where *M. loti* supplies vitamin B12, and in return, the bacterium receives fixed carbon from the microalga [45,46,47]. Mathematical models that analyze the nutrient exchange between the microorganisms showed that *M. loti* regulates the production of cobalamin according to the requirements of the microalga rather than simply releasing nutrients to the medium, in this way creating a true mutualistic interaction with *L. rostrata* [46]. Using quantitative isobaric tagging proteomics (iTRAQ), the proteome of *L. rostrata* growing axenically with the addition of exogenous vitamin B12 or in co-culture with *M. loti* was analyzed [47]. Five hundred and eighty-eight algal proteins were determined, with a higher number of enzymes related to amino acid biosynthesis found in the co-cultures than in the axenic cultures and with a total of 18 enzymes involved in both the degradation and biosynthesis of amino acids being elevated. By contrast, photosynthetic proteins and those of chloroplast protein synthesis were significantly lower in *L. rostrata* cells in co-culture. Despite the stability of this synthetic mutualism, the responses of *L. rostrata* show that the co-culture induces some stress in the microalga, and it must adjust its metabolism accordingly [47].

*Chlamydomonas reinhardtii* is a B12-independent microalga that encodes a B12-independent methionine synthase gene (METE) that is suppressed with the addition of exogenous B12. In another synthetic interaction with *M. loti*, it showed a reduction of METE expression, due to the supply of the vitamin by the bacterium [45].

On the other hand, few studies have addressed interaction through the exchange of thiamine; in co-culture, the thiamine producer *Pseudoalteromonas* sp. allows the growth of the marine thiamine auxotroph microalgae *Ostreococcus lucimarinus* [48], and the marine bacterium *Dinoroseobacter shibae,* isolated from the benthic dinoflagellate *Prorocentrum lima*, provides vitamins B1 and B12 to its dinoflagellate host [49]. Interaction of the green alga *Auxenochlorella protothecoides* with *E. coli* is based mostly on the *E. coli* provision of thiamine derivatives and degradation products. Metabolite production analysis showed that residual cell-free medium from axenic *E. coli* culture contained thiamine pyrophosphate and the thiamine precursor and degradation product, 4-amino-5-hydroxymethyl-2-methylpyrimidine (HMP). These compounds were found to promote the growth, lipid content, and glucose uptake of *A. protothecoides* [50]. In a synthetic mutualistic model created by our group between the green microalga *Chlorella* spp. and the microalgae growth-promoting bacterium (MGPB) *Azospirillum brasilense,* [37], Palacios et al. [51] found that in co-cultures of *C. sorokiniana* and *A. brasilense,* the two microorganisms produce and consume thiamine, with benefit to both partners. They found that conditions such as pH and light intensity affect the release of thiamine in the interaction. Another vitamin that seems to play a key role in the mutualistic interaction between these two microorganisms is riboflavin (vitamin B2), a cofactor in antioxidation and peroxidation. Lopez et al. [41] reported that *A. brasilense* produces riboflavin and lumichrome (a compound produced by the light degradation of riboflavin, which is also suggested as a plant-growth promoter), and both compounds affect the growth and metabolism of *Chlorella sorokiniana*.

Iron is an essential micronutrient required for metabolic activities; however, it is a limiting element in much of the world’s oceans [52]. In microalgae, iron is a cofactor in the photosystem I, and its deficiency can reduce the ability of the photosynthetic apparatus, affecting the rates of carbon fixation and growth [53]. Some bacteria can acquire iron through the secretion of siderophores, which are low-molecular-weight compounds with a high affinity for insoluble Fe (III). Siderophores scavenge iron from environmental stocks and then transport the ferric form back to the cells [54,55]. The cultivation of *Dunaliella bardawil* with *Halomonas* sp. isolated from algae cultures highlighted the siderophores’ key role in microalgal growth under iron deficiency. The bacteria increased Fe solubility, in this way enhancing its availability to the algae, which in turn significantly improved the algal growth rate [56]. In addition to increasing Fe availability, growth-promoting bacteria have been found to facilitate nitrogen assimilation, due to their organic nitrogen remineralization activity, which in turn enhances biomass accumulation [57]. Several species of *Marinobacter* associated with dinoflagellates produce vibrioferrin (VF), an unusual low-affinity dicitrate siderophore. VF contains two α-hydroxy acid groups and is highly sensitive to light [58]. In a synthetic interaction, co-cultures of the dinoflagellate *Scrippsiella trochoidea* and *Marinobacter* sp. were able to use iron from VF chelates in the dark; however, following in situ photolysis of the chelates, the uptake increases 70% for the bacterium and more than 20-fold for the microalga. In this way, the labile iron is utilized for algal photosynthetic fixation of carbon. Afterward, the fixed carbon is used by the bacteria for growth and is ultimately used to synthesize the siderophore VF [58].

Another metabolite exchange found to play a role in some mutualistic interactions is the phytohormone indole-3-acetic acid (IAA) synthesized and released by the bacterium, using tryptophan released by the microalga. In a study of bacterial consortia associated with cosmopolite marine diatoms, it was found that *Sulfitobacter* spp. synthesizes and secretes IAA, which promotes the growth of the diatoms, using diatom-secreted and endogenous tryptophan. The widespread incidence of this signal exchange in the oceans was corroborated by metabolic and metatranscriptomic analysis. Both metabolites act as signal molecules in a complex exchange of nutrients that includes diatom-excreted organosulfur molecules and bacterial-excreted ammonia [59]. 

A main mechanism that maintains the mutualism between *C. sorokiniana* and *A. brasilense* seems to be the exchange of signaling involved in the producing and releasing of IAA by the bacterium, using tryptophan and thiamine released by the microalga [60]. In co-culture, an increased activity of tryptophan synthase in *C. sorokiniana* and indole pyruvate decarboxylase (IPDC) in *A. brasilense* was observed. When *A. brasilense* was cultured in exudates of *C. sorokiniana*, increased expression of the *ipdC* gene and IAA production and release by *A. brasilense* were found only when tryptophan and thiamine were present in a synthetic growth medium. Similarly, Pagnussat et al. [61] found an active expression of the bacterial *ipdC* gene when the green microalgae *Scenedesmus obliquus* was co-cultivated with *A. brasilense* SP245. Nutritional conditions seem to modulate the interaction between these two microorganisms; in experiments when the interaction was supported by a nutrient-rich medium, production of both the signal molecules IAA and tryptophan was detected but not when this interaction began with nitrogen-free (N-free) or carbon-free (C-free) media [62]. The exchange of IAA and tryptophan between *A. brasilense* and the microalgae *Chlorella vulgaris, Scenedesmus obliquus,* and *C. reinhardtii* was corroborated by Choix et al. [63] in experiments under different concentrations (15%, 25%, and 35%) of CO_2_. In all concentrations, the bacterium and the microalga, respectively, produced AIA and tryptophan during all the experimental time, indicating that the synthesis of this compound is not affected by high CO_2_ concentrations.

**Figure 1:**
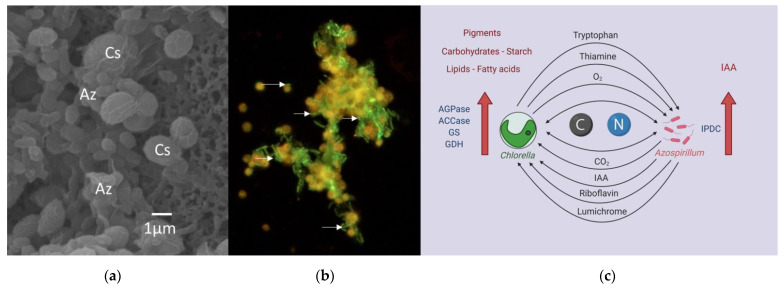
Synthetic mutualism between *Chlorella* spp. and the microalga growth promoting bacteria (MGPB) *Azospirillum brasilense*. (**a**) Scanning electron microscopy. (**b**) Epifluorescence microscopy. Auto-fluorescence of microalgae appears in orange while bacteria appear in green as result of fluorescent in situ hybridization (FISH) using three specific probes targeting Eubacteria (FAM dye) and one specific probe for *A. brasilense* (CY3 dye). Arrows show cells of *A. brasilense* attached to the microalga. (**c**) Model of the synthetic interaction. Az, *Azospirillum brasilense*; Cs, *Chlorella sorokiniana*.

Several studies have shown that *A. brasilense* affects the activities of key enzymes in metabolic pathways in *Chlorella* spp., resulting in the enhancement in production of metabolites of biotechnological importance, as is discussed later. Thus, there is an increase in activities of glutamine synthetase (GS: EC 6.3.1.2) and glutamate dehydrogenase (GDH, EC 1.4.1.3), leading to a higher uptake of nitrogen from the medium and a higher accumulation of intracellular nitrogen [64]. Similarly, there is an effect on ADP-glucose pyrophosphorylase (AGPase, EC 2.7.7.27), an enzyme that regulates starch biosynthesis, leading to an increased accumulation of starch [65], and on acetyl-CoA carboxylase (ACC, EC 6.4.1.2), a key enzyme in de novo fatty acid biosynthesis, increasing the synthesis of fatty acids and the accumulation of total lipids [66] (Figure 1).

Further demonstration of the mutualistic interaction between *C. sorokiniana* and *A. brasilense* was done by using stable isotope enrichment experiments followed by high-resolution secondary ion mass spectrometry (nanoSIMS) imaging of single cells. The transfer of carbon and nitrogen compounds between the two organisms was demonstrated by de-Bashan et al. [27]; the bacteria significantly enhanced the growth of the microalgae, while the microalgae supported the growth of the bacteria in a medium where it could not otherwise grow. Interestingly, similar to the findings of Helliwell et al. [47] in the synthetic mutualism between *L. rostrata* and *M. loti*, the physical attachment between *C. sorokiniana* and *A. brasilense* is not an obligate requirement for the establishment of an effective mutualistic association.

## 3. Enhancement of Biomass and Metabolite Production

The noteworthy increase in microalgae biomass production as a result of co-cultivation with bacteria has driven the study of these mutualistic interactions to boost the production of several microalgae of commercial interest (Table 1).

Some of the first studies have been focused on the synthetic mutualism described earlier between *Chlorella* co-cultivated with *A. brasilense*. These studies have described the positive effects on microalgae growth derived from this association, such as significant increments in cell size, biomass, growth rate, and productivity [70,75,79]. In other studies, the bacteria selected for co-cultivation have been isolated from wastewater effluents or the surrounding medium of the target microalgae species. For instance, Toyama et al. [32] found that growth-promoting bacteria were ubiquitously present in a wide variety of wastewater effluents. These isolated bacteria promoted cell growth of three different algae strains (*C. reinhardtii*, *C. vulgaris,* and *Euglena gracilis*), enhancing microalgal growth up to 2.8-fold. On the other hand, the bacteria *Pelagibaca bermudensis* isolated from the phycosphere of *Tetraselmis striata* enhanced two-fold the biomass productivity of this microalga [69], while population growth was 0.5–3 times higher in *Chlorella ellipsoidea,* with eight bacterial strains isolated from a long-term culture of *C. ellipsoidea* [68]. Growth-promoting bacteria were also found by Lee et al. [80] in the phycosphere of *Haematococcus pluvialis*. The authors reported an increase in *H. pluvialis* growth at all growth stages, due to high auxin production by co-cultivation with the isolated strain *Achromobacter* sp. CBA4603. Similarly, the co-cultivation of four bacterial strains (*Flavobacterium*, *Hyphomonas*, *Rhizobium,* and *Sphingomonas*) isolated from *C. vulgaris* increased the microalgal population by more than 100% when compared to axenic cultures [81]. Likewise, the growth-promoting effect of the marine bacterium *Flavobacterium* sp. was evaluated in three marine microalgae (*Chaetoceros gracilis*, *Isochrysis galbana*, and *Pavlova lutheri*). The results revealed that the bacterium enhanced the specific growth rate and maximal density of *C. gracilis* and kept longer the exponential growth phase of *I. galbana* and *P. lutheri* [82].

The cultivation of microalgae with growth-promoting bacteria results not just in the enhancement of biomass production but also in the increment of the intracellular levels of lipids, carbohydrates, pigments, and proteins (Table 2). Most of the recent studies on artificial microalgal–bacterial consortia are focused on lipid content, due to the increasing interest in biofuels and biodiesel production. The studies show that this kind of microbial association improves both lipid productivity and lipid quality for biodiesel production. For instance, lipid accumulation in the microalga *C. reinhardtii* was significantly improved by co-cultivation with *Azotobacter chroococcum* under nitrogen starvation [83]. The authors reported an increase of 2.4 times in lipid content and 5.9 times in lipid production with the co-culture and up to 19.4 times the lipid productivity compared with the axenic microalga. This increment was explained by an increase in the levels of expression of genes that positively regulated lipid metabolism, while the expression levels of genes that negatively regulated lipid metabolism decreased. Similarly, Leyva et al. [66] found that the activity of acetyl-CoA carboxylase (ACCase) is enhanced by the co-cultivation of *C. vulgaris* with the MGPB *A. brasilense* under autotrophic and heterotrophic conditions. However, although higher levels of lipids were found in co-cultures (up to a five-fold increase under autotrophic conditions), the authors did not find a direct link with the increase on ACCase activity. Likewise, the total content of C16 and C18, which are the main fatty acids present in biodiesel composition, can increase in symbiotic co-cultures. Xue et al. [83] reported more than 80% content of C16 and C18 in the fatty acids produced by the microalga *C. vulgaris* when cultivated with *Stenotrophomonas maltophilia* as well as an increase of up to 5% when compared to axenic cultures. Similar results were reported by de-Bashan et al. [75] with the cultivation of three different *Chlorella* strains with *A. brasilense* immobilized in alginate beads. Immobilization has been found to maintain the close physical proximity of the two microorganisms to facilitate interaction and avoid external interference from bacterial contaminants [84]. In all the co-cultures, the concentration and variety of fatty acids increased, reaching up to eight different fatty acids in microalgae co-immobilized with the MGPB in comparison to four to five in microalgae-only immobilized cells.

Although less attention has been paid to the production of microalgal pigments, carbohydrates, and proteins by co-cultivation with bacteria, a few studies have also revealed the ability of these bacteria to promote the production of these metabolites in microalgae. For instance, the cultivation of *A. brasilense* with the microalgae *S. obliquus*, *C. vulgaris,* and *C. reinhardtii* under high CO_2_ concentrations, as discussed earlier, significantly enhanced microalgal growth as well as metabolite accumulation on each microalga. The authors reported an increase of carbohydrates, proteins, and lipids under all gas mixtures evaluated [63]. Higher levels of microalgal carbohydrates have also been reported as a result of co-cultivation with bacteria. Higgins and VanderGheynst [72] reported significant increments in the starch produced by *Chlorella minutissima* when co-cultured with *E. coli* under mixotrophic conditions (glucose, glycerol, and acetate substrates). At 1% substrate concentration, the total starch productivity as well as lipid productivity increased in all the co-cultures compared to axenic conditions. The co-cultivation of two *Chlorella* strains (*C. vulgaris* and *C. sorokiniana*) with *A. brasilense* supports the positive effect of the bacteria on carbohydrate production of this microalgae genus [70,79]. In these studies, the authors reported up to 72% and 90% increments, respectively, in total carbohydrates of *C. vulgaris* under autotrophic and heterotrophic conditions, while *C. sorokiniana* had an increase in carbohydrate production of around 55% under autotrophic conditions and 21% under heterotrophic conditions.

Similarly, microalgal pigment production can be significantly enhanced by co-cultivation with bacteria. Gonzalez-Bashan et al. [88] found that the production of chlorophyll, ß-carotene, lutein, and violaxanthin increased significantly in the microalga *C. vulgaris* when grown with *Phyllobacterium myrsinacearum* co-immobilized in alginate beads. A similar study was carried out with *A. brasilense*, enhancing even more the pigment production of *C. vulgaris*. The co-immobilization of the microorganisms resulted in increments of up to 35% in chlorophyll a, 176% in chlorophyll b, 186% in ß-carotene, 152% in lutein, and 129% in violaxanthin [87]. Likewise, a significant increase in these four pigments was observed in the microalga *C. sorokiniana* when co-immobilized in alginate beads with *A. brasilense* [75]. The use of growth-promoting bacteria to enhance pigment production has great commercial potential, considering the increasing consumer demand for natural products, including the replacement of commonly used synthetic pigments for pigments derived from natural sources [89].

## 4. Green Chemistry Projections and Circular Bioeconomy

**Figure 2:**
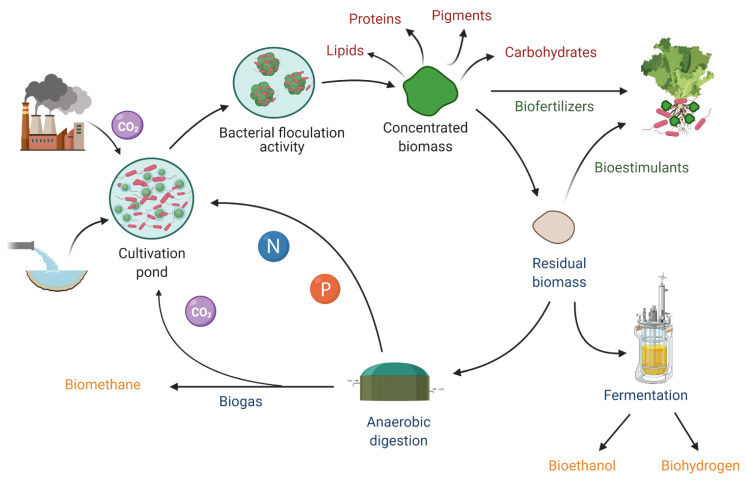
Schematic model of a microalgae–bacteria sustainable and cost-efficient system for the production of several metabolites within a biorefinery using different biological routes.

The development of sustainable and cost-efficient systems for microalgal metabolite production is a key area of research, due to the increasing demand by consumers around the world for natural and sustainable labeled products. The cost of production of microalgae-based proteins, lipids, pigments, and carbohydrates is still a bottleneck that prevents many industries from making this desirable shift. One way to overcome this issue is to integrate metabolite production in a biorefinery that maximizes the biomass value while reducing overall costs. The co-cultivation of microalgae with growth-promoting bacteria plays a key role in the optimization of microalgae biorefineries (Figure 2).

The integration of microalgae production systems for the utilization of mass and energy is widely explored through algae-based biorefineries [90,91,92], which involve consecutive physical and chemical fractionation steps to recover different microalgal components (proteins, lipids, carbohydrates, and pigments). The order of extractions depends on the value and sensitivity of the target products [93]. Although, as we described earlier, growth-promoting bacteria can significantly enhance the production of these metabolites; to the authors’ knowledge, no studies are evaluating microalgae–bacteria consortia in these kinds of biorefineries. Most of the studies that use these consortia integrate wastewater treatment with biofuel production in an attempt to reduce the high cultivation cost of microalgal biomass while at the same time removing nutrients and pollutants from wastewater [94]. The use of wastewater as the culture medium for microalgae may reduce the use of commercial fertilizers and at the same time minimize freshwater consumption [95,96,97]. Therefore, the use of microalgae–bacteria consortia in wastewater treatment has been widely studied, revealing that the microbial community plays an important role on the performance of the system [98,99,100]. Because the natural bacterial communities in wastewater effluents are a combination of beneficial and competing species, artificial consortia are preferred to boost microalgal biomass production. For instance, analyses of the microbiome associated with the microalga *Chlorella sorokiniana* cultivated on anaerobic digestate effluent revealed that the microalgae biomass was correlated negatively with the relative abundance of the genus *Pusillimonas* [98]. Conversely, a significant improvement on microalgal biomass was observed with an artificial consortium comprised of two *Chlorella* strains and the bacteria *Klebsiella pneumoniae* and *Acinetobacter calcoaceticus* growing in artificial wastewater as well as in raw dairy wastewater [101]. Furthermore, several studies of wastewater treatment with microalgae and MGPB co-immobilized in alginate beads have revealed the positive effect of the bacteria on microalgal growth, metabolism, and nutrient removal [75,88]. However, the production of lipids integrated with wastewater treatment is focused mostly on microalgae and bacterial communities instead of on artificial microalgae–bacteria one-to-one consortia. Tighiri and Erkurt [102] demonstrated the efficiency of microalgae–bacteria consortia in leachate treatment and biomass production for biorefinery purposes. The authors reported 100% removal of ammonium and up to 90% removal efficiencies for nitrate, chemical oxygen demand, and phenol. Furthermore, the relative toxicity was reduced from 57.32 to 1.12%, while the fatty acid content (C16–18) reached up to 88% of the biomass. Bélanger-Lépine et al. [103] evaluated lipid production from an alga–bacteria consortium grown in three different wastewaters and leachates in comparison with the consortium grown in a commercial medium. The results showed that growth and lipid production was the same or higher when compared to the standard medium, which implies significant savings in the production process. Likewise, Chen et al. [104] compared the ability of two microalgae strains (*Chlorella* sp. and *Scenedesmus* sp.) and the microbial community of activated sludge alone or in combination to produce lipids and remove nutrients from artificial municipal wastewater under light/dark conditions. The results showed greater advantages with the symbiotic systems compared with the sterile ones.

On the other hand, the production of other metabolites of commercial interest, such as proteins, carbohydrates, and pigments, by these microalgae–bacteria biorefineries is worth exploring, since it can give additional value to the harvested biomass while lowering the cost of production. Moreover, in addition to the production of these valuable products, the residual biomass can be used for biogas [105,106,107], bioethanol [108,109], or biohydrogen production [110,111]. For instance, in the biorefinery proposed by Nobre et al. [112], the species *Nannochloropsis* sp. was studied for the production of oils and pigments as well as for biohydrogen production from the leftovers. With symbiotic systems, Lakatos et al. [113] reported a significant enhancement of hydrogen production from the microalgae *Chlamydomonas* sp. MACC-549 and *C. reinhardtii* cc124 when co-cultivated with bacterial symbionts. The bacterial strains enhanced hydrogen production by reducing the oxygen level. The maximum hydrogen yield was found with the cultivation of the hydrogenase-deficient *E. coli*. Likewise, several studies integrate lipid production from microalgae grown in wastewater [114,115] and the use of the lipid spent biomass for methane production through anaerobic digestion [105,106,116,117]. The use of microalgae–bacteria consortia in these biorefineries is gaining more attention with respect to increasing microalgal productivity while improving economic feasibility [9]. For instance, Bohutskyi et al. [118] evaluated the lipid production in an alga–bacteria polyculture grown in municipal primary wastewater and also evaluated the biomethane potential of the lipid extracted residues. The harvested biomass contained around 23% lipid content, including fatty acid methyl esters optimal for biodiesel production. Although methane production of the lipid-extracted biomass was low (296 ± 2 mL/gVS), the biomethane potential was maximized by hydrothermal liquefaction pretreatment (to increase biomass biodegradability) and co-digestion with sewage sludge (to reduce toxicity due to solvent residues). The anaerobic digestion of microalgae biomass leftovers is a sustainable way to increase the overall efficiency of biorefineries, because not only is energy recycled (biomethane is burned to produce the electricity required to maintain the system), but nutrients and CO_2_ can also be recycled in the culture pond [119,120]. To improve methane production and the general extraction of the targeted metabolites, it is important to select microalgae species with thin and/or weak cell walls to facilitate extraction and to improve their biodegradability [119,121]. On the other hand, wet extractions could eliminate the inhibition in the anaerobic digestion process caused by organic solvents and at the same time significantly reduce the cost and energy input involved in drying the biomass [105,122].

One interesting way in which bacteria can improve microalgae biorefineries is through the selection of bacterial species with high flocculation activity to optimize the harvesting process, which is one of the biggest challenges, since it accounts for a considerable part of the energy and costs invested in the biorefinery process [123]. Powell and Hill [124] explored this way of aggregating algae by cultivating *Nannochloropsis oceanica* with the bacterium *Bacillus* sp., reaching up to 95% efficiency, with aggregates formed in 30 s. Another bacterium, designated as strain HW001, was capable of aggregating several microalgae and cyanobacteria species, including potential biofuel-producing microalgae, such as *N. oceanica*, without affecting the lipid content [14,125]. Likewise, Cho et al. [81] cultivated *C. vulgaris* with isolated bacteria from the same culture and observed that the artificial microalgae–bacteria consortia had flocculation efficiency, lipid content, and quality superior to that of the axenic microalgae culture. The flocculation of *C. vulgaris* by co-cultivation with its associated bacteria was also evaluated by Lee et al. [126]. The flocculating activity reached up to 94% with the co-cultures, compared to just 2% achieved with the axenic culture.

Microalgal biorefineries can also be optimized through bacterial cell-free extracts. For instance, the cultivation of the microalgae *Characium* sp. in the cell-free filtrate of *Pseudomonas composti* resulted in an increase of 57% in the microalgal biomass, while lipid productivity increased by 18% [40]. Furthermore, enzymatic extracts of biomass-degrading bacterial strains can assist in the hydrolysis of the microalgal cell walls facilitating metabolites extraction. Guo et al. [127] reported 40% of cell disruption in cultures of the microalga *Chlorella zofingiensis* with the enzymatic extract of the bacterium *Bacillus* sp, which allowed wet lipid extraction. Likewise, a nearly 100% increase in lipid extraction efficiency was reported with the cultivation of *Chlorella vulgaris* with an enzymatic extract of *Flammeovirga yaeyamensis* [128]. Similarly, remote effects of bacteria-derived volatiles (mainly CO_2_, 2,3-butanediol, and acetoin) positively affected growth and metabolism in the microalga *C. sorokiniana*. The MGPB *A. brasilense* and *Bacillus pumilus* remotely enhanced microalgal growth as well as lipids, carbohydrates, and chlorophyll a production [42]. Although in a microalga–bacteria biorefinery the target metabolites are derived from the co-culture biomass, the use of bacterial exudates or volatiles might overcome the challenge of separating the microalgal and bacterial biomasses when it is required. 

A possible route to explore to improve biorefinery sustainability is the use of the residual biomass as a biostimulant in food crops to reduce the use of chemical fertilizers [129,130,131]. There is an increased interest in sustainable agriculture through the replacement of traditional synthetic fertilizers by environmentally friendly biofertilizers and biostimulants [132]. Valuable compounds from microalgae–bacteria biomass residues can be utilized as an amendment for soils. Microalgae contain some plant-growth-promoting substances, such as amino acids, phytohormones (auxins, cytokinins, abscisic acid, ethylene, and gibberellins), polyamines, betaines, vitamins, protein hydrolysates, and polysaccharides that can be used as biostimulants [132,133]. Likewise, the high potential of plant-growth-promoting bacteria as biofertilizers is well documented, due to their ability to promote growth in plants [134]. The efficiency of alginate beads containing *C. sorokiniana* and *A. brasilense* has been evaluated as an amendment for infertile soils having low levels of organic matter. After drying the beads, the bacteria survived for one year. The application of the dry beads increased organic matter, organic carbon, and microbial carbon in the soil. Growth of sorghum in the amended soil was greater than that of plants grown in low organic matter, untreated soil, or soil amended with beads of just alginate or containing only microalgae or bacteria. The surface of the plant roots growing in the amended soil was heavily colonized by *A. brasilense*. Application of the beads significantly changed the rhizosphere bacterial population structure, and species richness and diversity increased with the beads containing the microalgae–bacteria association [135,136]. Likewise, Raposo and Morais [137] evaluated three rhizobacteria–microalgae consortia encapsulated in microbeads of different combinations of maltodextrin, arabic gum, and gelatine as a soil amendment for meadow clover plantlets. The authors found that *Pseudomonas putida*, *Serratia proteomaculans* and *Stenotrophomonas maltophilia* in consortia with the microalgae *Chlorella vulgaris* enhanced plantlets’ growth by improving their root and leaf area. The viability of bacteria after metabolite extraction depends on the target metabolite and the extraction process. For instance, wet solvent-free lipid extractions [105] would be a suitable alternative to preserve the bacterial activity after lipid extraction. Nevertheless, even when the extraction process prevents bacterial survival, the residual biomass can be used as an organic-rich amendment. Likewise, one fraction of the co-culture can be used directly as soil fertilizer (Figure 2) giving an added value to the biorefinery.

Another way to improve the sustainability and economic feasibility of the biorefinery is by dropping the CO_2_ supply. The non-negligible cost of production related to microalgae CO_2_ consumption can be significantly reduced, not just through the CO_2_ generated from the bacterial partner but also by using CO_2_ emissions from industrial plants in the cultivation systems [138,139] or through biogas upgrading [140]. This is a practical way of lowering costs while helping in the mitigation of CO_2_ emissions. Although high CO_2_ concentrations can be detrimental to microalgal physiological activity and hence CO_2_ sequestration [141], growth-promoting bacteria can play a key role in improving CO_2_ capture by microalgae. As presented elsewhere, Choix et al. [63] evaluated the CO_2_ fixation rate of three microalgae species in co-culture with *A. brasilense* under different CO_2_ concentrations. The results revealed that *A. brasilense* has the ability to mitigate the stress caused by CO_2_ levels of up to 35%, improving the CO_2_ fixation rate by 25–50% in *S. obliquus*, 46–87% in *C. vulgaris,* and 41–53% in *C. reinhardtii*. Besides, as stated before, production costs associated with fertilizer consumption can be minimized either by using wastewater as the culture medium, or by recycling the main nutrients, such as nitrogen and phosphorous recovered after anaerobic digestion [142,143,144] or hydrothermal liquefaction conversion [145,146]. The successful development of solar-driven industries would be a big step toward the maintenance of the earth system between the planetary boundaries’ frameworks [147].

## 5. Concluding Remarks

In summary, a successful microalgae-bacteria consortia must be beneficial to all microorganisms. The demonstration of mutual benefits and the association’s self-maintenance duration over several consecutive generations is fundamental to the validation of this approach. Knowing each of the partners’ specific nutritional and environmental requirements can help the manipulation of the intended biotechnological response. Thus, although genomic, transcriptomic, and metabolomic studies in synthetic mutualistic consortia are currently very limited, they are of utmost importance in understanding the underlying mechanisms acting in establishing and stabilizing the mutualism.

The development of microalgal biorefineries based on mutualistic interaction between growth-promoting bacteria and microalgae stands as an attractive alternative in overcoming the drawbacks that currently jeopardize the application of these biorefineries on a large scale. Currently, the development of sustainable and cost-efficient systems for microalgae metabolite production is a key area of research, due to the increasing demand by consumers around the world for natural labeled products. 

Lastly, the development of circular economy principle-based systems, such as microalgae–bacteria consortia biorefineries, constitutes an option to reduce CO_2_ emissions within the framework of meeting the international COP21 Paris CO_2_ emission reduction commitments and UN Sustainable Development Goals [148].

## Figures and Tables

**Table 1:** Microalgal growth promotion of some artificial microalgae–bacteria consortia.

Bacteria	Microalgae	Growth Promotion Effect	Culture Medium	Reference
*Azospirillum brasilense* Cd	*Chlorella sorokiniana* UTEX 2714	11% increase in cell density (g dw/L)	N8 medium	[67]
*Azospirillum brasilense* Cd	*A. protothecoides* UTEX 2341	90% increase in cell density (g dw/L)	N8-NH_4_	
*Brevundimonas* sp.	*Chlorella ellipsoidea* UTEX 247	50-fold increase in cell density (cel/mL), longer exponential phase	Modified BBM	[68]
*Pelagibaca bermudensis* KCTC 13073BP	*Tetraselmis striata* KCTC1432BP	2-fold increase in biomass productivity (mg/L/d)	O3 medium	[69]
*Azospirillum brasilense* Cd	*Chlorella vulgaris* UTEX 2714	16 and 11% increase in cell density (cel/mL) and growth rate, respectively	Synthetic growth medium (SGM)	[70]
*Azospirillum brasilense* Cd	*Chlorella sorokiniana* UTEX 2805	40 and 35% increase in cell density (cel/mL) and growth rate, respectively		
*Bacillus pumilus* ES4	*Chlorella vulgaris* UTEX 2714	1.5-fold increase in cell density (cel/mL)	N-free SGM	[71]
*Escherichia coli* ATCC 25922	*Chlorella minutissima* UTEX 2341	3.5-fold biomass productivity (mg/L/d)	N8-NH_4_, 1% Glucose	[72]
		3.4-fold biomass productivity (mg/L/d)	N8-NH_4_, 1% Glycerol	
		7.2-fold biomass productivity (mg/L/d)	N8-NH_4_, 1% Acetate	
*Rhizobium* sp. 10II	*Ankistrodesmus* sp. SP2-15	29% increase in dry weight (mg/L)	BG11 medium	[73]
*Stenotrophomona smaltophilia*	*Chlorella vulgaris*	22, 20, and 18% increase in biomass (g/L), growth rate and productivity (mg/L/d), respectively	BG11 medium	[74]
*Azospirillum brasilense* Cd	*Chlorella vulgaris* UTEX 395	62% increase in cell size	Synthetic wastewater	[75]
*Azospirillum brasilense* Cd	*Chlorella vulgaris* UTEX 2714	3-fold increase in cell density		
*Azospirillum brasilense* Cd	*Chlorella sorokiniana* UTEX 1602	2.2-fold increase in cell density		
*Rhizobium* sp.	*Botryococcus braunii*	55% increase in optical density	Modified Jaworski medium	[33]
*Muricauda* sp.	*Dunaliella* sp.	7% increase in cell biovolume	Modified Walne’s medium	[57]
*Dinoroseobacter shibae*	*Thalassiosira* *pseudonana*	35% increase in cell density	SW+ medium	[76]
*Phaeodactylum tricornutum*	*Stappia* sp.	72% increase in cell density	F/2 medium	[77]
*Alteromonas* sp.	*Isochrysis galbana*	52% increase in cell density	Zobell Marine Broth	[78]
*Labrenzia* sp.	*Isochrysis galbana*	71% increase in cell density		

**Table 2:** Metabolite production enhancement of some artificial microalgae–bacteria consortia.

Bacteria	Microalgae	Metabolite Production Enhanced	Culture Medium	Reference
*Escherichia coli* ATCC 25922	*Chlorella minutissima* UTEX 2341	6.2-fold lipid productivity (mg/L/d)	N8-NH_4_, 1% Glucose	[72]
		18.8-fold starch productivity (mg/L/d)		
		1.8-fold lipid content (%)		
		5.4-fold starch content (%)		
		3.1-fold lipid productivity (mg/L/d)	N8-NH_4_, 1% Glycerol	
		9.9-fold starch productivity (mg/L/d)		
		2.9-fold starch content (%)		
		8.2-fold lipid productivity (mg/L/d)	N8-NH_4_, 1% Acetate	
		27.1-fold starch productivity (mg/L/d)		
		3.7-fold starch content (%)		
*Azotobacter chroococcum* No 1.0233	*Chlamydomonas reinhardtii* cc849	2.4-fold lipid content (%)	N-free TAP medium	[83]
		5.9-fold lipid production (mg/L)		
		19.4-fold lipid productivity (mg/L/d)		
*Stenotrophomona smaltophilia*	*Chlorella vulgaris*	Lipid increase by 8–34%	BG11	[74]
*Phaeodactylum tricornutum*	*Stappia* sp.	172% increase in fucoxanthin	F/2 medium	[77]
		144% increase in chlorophylls		
*Phaeodactylum tricornutum*	*Marinobacter* sp.	50% increase in total lipids	F/2 medium	[85]
*Rhizobium* sp. 10II	*Ankistrodesmus* sp. SP2-15	39% increase in chlorophyll a	BG11	[73]
*Methylococcus capsulatus*	*Chlorella sorokiniana*	42% increase in carbohydrates	Industrial wastewater with synthetic biogas as methane source	[86]
*Methylococcus capsulatus*	*Chlorella sorokiniana*	15% increase in lipid content		
*Azospirillum brasilense* Cd	*Chlorella sorokiniana* UTEX 1602	1.6-fold chlorophyll a (µg/g cells)	Synthetic Wastewater	[75]
		1.6-fold chlorophyll b (µg/g cells)		
		1.7-fold lutein (µg/g cells)		
		2.5-fold violaxanthin (µg/g cells)		
		5.5-fold lipid content (µg/g dw)		
*Azospirillum brasilense* Cd	*Chlorella vulgaris* UTEX 395	1.6-fold chlorophyll a (µg/g cells)		
		1.8-fold chlorophyll b (µg/g cells)		
		1.8-fold lipid content (µg/g dw)		
*Azospirillum brasilense* Cd	*Chlorella vulgaris* UTEX 2714	2.8-fold chlorophyll a (µg/g cells)		
		2.5-fold chlorophyll b (µg/g cells)		
		2.3-fold lutein (µg/g cells)		
		1.5-fold violaxanthin (µg/g cells)		
		3.9-fold lipid content (µg/g dw)		
*Azospirillum brasilense* Cd	*Chlorella vulgaris* UTEX 2714	1.4-fold chlorophyll a (µg/g cells)	Synthetic Wastewater	[87]
		2.8-fold chlorophyll b (µg/g cells)		
		2.9-fold ß-carotene (µg/g cells)		
		2.5-fold lutein (µg/g cells)		
		2.3-fold violaxanthin (µg/g cells)		
*Phyllobacterium* *myrsinacearum*	*Chlorella vulgaris* UTEX 2714	1.8-fold chlorophyll b (µg/g cells)	Synthetic Wastewater	[88]
		1.8-fold ß-carotene (µg/g cells)		
		2-fold lutein (µg/g cells)		
		2.2-fold violaxanthin (µg/g cells)		
*Azospirillum brasilense* Cd	*Chlorella sorokiniana* UTEX 2714	3-fold chlorophyll a (µg/mg dw)	N8 medium	[67]
		5-fold chlorophyll b (µg/mg dw)		
		2.5-fold soluble protein (%)		
*Azospirillum* *brasilense* Cd	*A. protothecoides* UTEX 2341	40–60% increase in soluble protein	N8-NH_4_	

## Data Availability

Not applicable.
